# One virus—many strategies: type-specific interactions between human adenoviruses and innate immunity

**DOI:** 10.1128/jvi.00204-26

**Published:** 2026-08-03

**Authors:** Lisa Hanrieder, Sabrina Schreiner

**Affiliations:** 1 School of Medicine, Institute of Virology, Technical University of Munich 155892, Munich, Germany; 2 Institute of Virology, Medical Center - University of Freiburg 9174, Freiburg, Germany; 3 Institute of Virology, Hannover Medical School 686461, Hanover, Germany; 4 Cluster of Excellence RESIST (Resolving Infection Susceptibility; EXC 2155), Hannover, Germany; New York University Department of Microbiology, New York, New York, USA

**Keywords:** AdV, types, innate immunity, interferon, IFN

## Abstract

Human adenoviruses (HAdVs) are double-stranded DNA viruses that can cause a wide range of infections, including respiratory, gastrointestinal, and ocular diseases, as well as less common conditions such as hepatitis, hemorrhagic cystitis, and encephalitis. While most infections in immunocompetent individuals remain clinically asymptomatic, immunocompromised patients are at a high risk of developing severe HAdV infections. Due to their exceptional transduction efficiency, HAdVs are also widely used as vaccine vectors, such as in vector-based COVID-19 vaccines. Although HAdVs are present as pathogens and vectors, the interaction between HAdVs and the human immune system remains insufficiently studied. Most adenoviral basic research in this context has focused on HAdV-C5. However, differences in capsid structure and genome organization exist not only between adenovirus species but also among types within the same species. These variations may influence the ability of the virus to evade or counteract the host immune system, ultimately influencing viral infectivity and replication efficiency. Further studies are needed to elucidate these differences. In this review, we summarize the differences in immune responses, with a particular emphasis on triggered IFN responses, across various HAdV species and their influence on infection outcomes, as well as their implications for the use of adenoviral vectors. Future research addressing these differences will be essential to better understand adenovirus pathogenesis and the rational design of adenovirus-based vectors.

## INTRODUCTION

### Classification, pathology, and virion structure of human adenoviruses

Human adenoviruses (HAdVs) are 80–110 nm large non-enveloped viruses containing a non-segmented linear double-stranded DNA (dsDNA) genome with around 36 kb (1). They belong to the Adenoviridae family and are classified into seven different species A to G, each comprising multiple types. These types are primarily distinguished based on their phylogenetic relationships, genome organization, tissue tropism, and immunological cross-neutralization (1–3). The variations in tissue tropism among HAdV types result in diverse clinical manifestations, ranging from respiratory, gastrointestinal, and ocular infections to less common conditions such as hepatitis, hemorrhagic cystitis, and encephalitis. In immunocompetent individuals, HAdV infections are typically mild and self-limiting. However, in immunocompromised patients—whether due to congenital conditions, acquired immunodeficiencies, or drug-induced immunosuppression—HAdV infections can result in severe and even fatal outcomes (4).

Adenoviral species A, F, and G predominantly infect the gastrointestinal tract, species B, C, and E primarily target the respiratory system, while species D is mainly associated with ocular infections (1). The mechanisms underlying the different tissue tropisms of adenoviral species are not yet fully understood. Differences in capsid organization, receptor usage during virus entry, and subsequent antiviral host strategies have been suggested as possible explanations for these observations (5).

The icosahedral adenoviral capsid is composed of three major structural proteins: hexon, penton, and fiber proteins, accompanied by four minor capsid proteins: IIIa, VI, VIII, and IX (6, 7). The HAdV genome is structured into distinct transcriptional units, including early (E1A, E1B, E2A, E2B, E3, and E4), intermediate (pIX, IVa2, and L4), and late (major late transcription unit [MLTU]) units. The early and intermediate regions encode regulatory proteins involved in host and viral gene expression as well as factors required for viral DNA replication. These functions contribute to activation of the major late promoter (MLP). This promoter initiates, along with accumulating viral DNA, the transcription of late-expression units. These late units are subsequently processed via alternative splicing and are responsible for encoding the structural proteins required for the assembly of new virions (8–10).

Beyond their role as pathogens, HAdVs have been widely utilized as vectors for vaccine development, including in SARS-CoV-2 and Ebola vaccines. Their high transduction efficiency, capacity for high-titer production, and inherent ability to induce moderate levels of innate immunity without the need for adjuvants make HAdVs highly attractive platforms for vaccine delivery (11).

### Key players of the human immune system

The adenoviral capsid and DNA components both separately trigger host immune signaling pathways, with outcomes differing based on the adenovirus species and the type of infected cell. In general, antiviral host defense can be divided into innate immunity, including intrinsic immunity, and adaptive immunity (12, 13). Innate immunity relies on germline-encoded pattern recognition receptors (PRRs) that detect conserved pathogen-associated molecular patterns (PAMPs) and rapidly initiate antiviral responses. In this context, intrinsic antiviral immunity represents a cell-autonomous component of innate immunity in which preexisting restriction factors directly limit viral replication and assembly within infected cells (13). In contrast, adaptive immunity is mediated by antigen-specific receptors on B and T lymphocytes and generates highly specific responses, as well as a long-lasting immunological memory (12, 14, 15).

Cellular components of the innate immune system include macrophages and neutrophil granulocytes, natural killer cells, antigen-presenting dendritic cells, and epithelial cells (14). These cells utilize a variety of PRRs, including Toll-like receptors (TLRs), Nod-like receptors, and scavenger receptors, which detect PAMPs such as components of viral or bacterial envelopes or foreign nucleic acids. For example, the endosomal receptor TLR9 detects foreign DNA sequences, whereas cytosolic sensors such as RIG-I recognize viral RNA and such as cGAS detect cytosolic DNA. PRRs can also detect damage-associated molecular patterns (DAMPs), which are released during tissue damage (14, 16–19).

**Fig 1:**
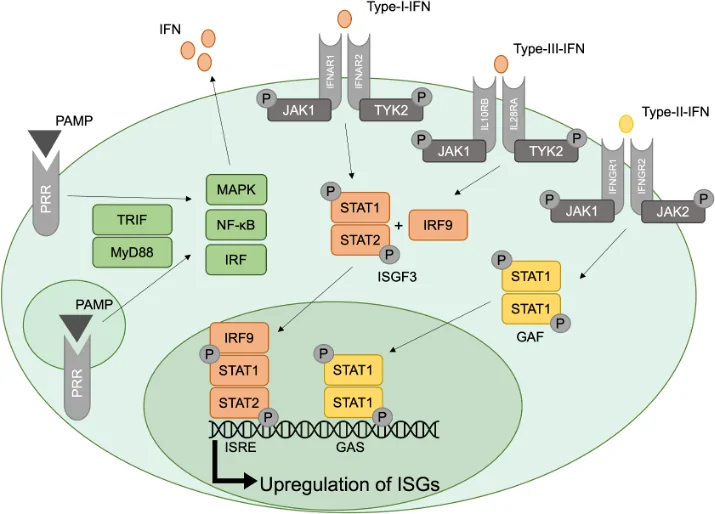
Schematic overview of the IFN signaling pathway. PAMPs activate a PRR-initiated pathway, resulting in IFN secretion. IFNs interact with their specific receptor and lead to the activation of downstream signaling cascades, resulting in the upregulation of ISGs (14, 16–20). PAMP = pathogen-associated molecular patterns, PRR = pattern recognition receptor, TRIF = TIR-domain-containing adapter protein inducing interferon-β, MyD88 = myeloid differentiation primary response 88, MAPK = mitogen-activated protein kinase, NF-κB = nuclear factor kappa B, IRF = interferon-regulating factor, STAT = signal transducer and activator of transcription, IRSE = interferon-stimulated response element, GAS = gamma interferon-activated site, ISG = interferon-stimulated gene, ISGF3 = interferon-stimulated gene factor 3, JAK = janus-activated kinase, TYK = tyrosine kinase, IFN = interferon, IFNAR1/2 = IFNα receptor 1/2, IL10RB/28RA = interleukin 10/28 receptor B/A, and IFNGR1/2 = IFNγ receptor 1/2.

Following ligand binding, PRR signaling cascades are activated, most commonly through the adapter proteins MyD88 or TRIF, leading to the activation of nuclear factor kappa B (NF-κB), mitogen-activated protein kinases (MAPKs), and interferon regulatory factors (IRF1, IRF3, IRF5, and IRF7). These signaling pathways ultimately result in the secretion of inflammatory cytokines such as interleukins, interferons (IFNs), and tumor necrosis factors, which amplify antiviral immune responses (14, 19, 20), (Fig. 1). In epithelial cells, these pathways primarily induce antiviral states through IFN signaling, whereas in professional immune cells such as dendritic cells and macrophages, they promote cytokine secretion and antigen presentation, thereby linking innate and adaptive immune responses (19, 21, 22).

IFNs are subdivided into three groups: Type-I IFNs, which include IFNα, β, κ, and ε/τ; Type-II IFNs, containing only IFNγ; and Type-III IFNs, including IFNλ1, λ2, and λ3 (23–26). Type-I IFNs bind to their corresponding transmembrane IFNα receptor (IFNAR), consisting of the two subunits IFNAR1 and IFNAR2, and are present on most cell surfaces (21, 27). The binding mainly induces receptor-associated protein tyrosine janus activated kinase 1 (JAK1) and tyrosine kinase 2 (TYK2) to recruit and phosphorylate the cytoplasmic transcription factors signal transducer and activator of transcription 1 (STAT1) and STAT2. Phosphorylated STAT1 and STAT2 form a heterodimer, which translocates into the nucleus and colocalizes with IRF9 (21, 28, 29). This complex is called IFN-stimulated gene factor 3 (ISGF3) and binds to an interferon-stimulated responsive element (ISRE) in the promoter region of interferon-stimulated genes (ISGs). This binding directly upregulates the transcription of ISGs, such as *cGAS*, *STING*, *OAS1,* and *IFITM1* (30).

Type-III-IFNs, λ1, λ2, and λ3 (also known as IL-28A, IL-28B, and IL-29), are encoded by three distinct genes. These IFNs interact with a receptor composed of two subunits, IL-28R1 and IL-10R2, which also initiates the formation of the ISGF3 complex. Subsequently, both Type-I- and Type-III-IFNs induce a similar pattern of ISG expression (26, 31–33).

IFNγ, in contrast, binds to the IFNGR1 and IFNGR2 subunits, which activate JAK1 and JAK2. These tyrosine kinases lead to phosphorylation of mainly STAT1. Phosphorylated STAT1 molecules form homodimers called the gamma-activated factors (GAFs), which translocate into the nucleus and bind to the gamma-activated sequences (GASs), located in the promoter region of specific ISGs (34, 35) (Fig. 1).

In this review, we summarize the differences in host immune responses, with a particular focus on innate IFN responses, across various HAdV species and their influence on infection outcomes, as well as their implications for the use of adenoviral vectors. In general, during viral infections, productive viral replication results in a lytic infection, including the release of viral progeny through cell lysis, tissue damage, and early apoptosis of the host cell (36, 37). In contrast, insufficient viral replication may lead to viral persistence, latency, or clearance of the infection. The viral proliferation and the host cell survival must be balanced by the virus to ensure optimal conditions for each virus. Strategies, therefore, include the regulation of host cell apoptosis, suppression of IFN production, modulation of MHC class I/II and therefore of cytotoxic lymphocyte and natural killer cell responses, and limitation of cytokine or chemokine production (38–40). HAdV primarily targets and inhibits cellular pathways involved in antiviral defense, cell growth arrest, and apoptosis, while reprogramming host cell metabolism to support efficient viral replication and production of viral progeny (41, 42). However, the dimensions of these strategies vary significantly across adenoviral types, leading to different courses and outcomes of infection.

## EARLY IMMUNE SIGNALING FOLLOWING ADENOVIRUS CELL ENTRY

The entry of HAdVs into host cells is facilitated by various host cell receptors, including Coxsackie and Adenovirus Receptor (CAR), CD46, desmoglein-2 (DSG-2), and glycans such as polysialic acids and GD1a (43–47). The employed cell receptors vary depending on the types, primarily due to distinct binding sites on the fiber knob and the length and flexibility of the fiber shaft of each adenoviral type (48, 49).

For viral entry, the HAdV fiber knob binds to its specific host cell receptor, whereas the HAdV penton base interacts with the cellular RGD-binding integrins, resulting in integrin-mediated endocytosis (50, 51). Once inside the host cell, the viral particles are transported toward the nucleus along microtubules and are imported into the nucleus (50). The endocytosed adenoviral capsid and DNA components activate several innate immune pathways, resulting in the upregulation of *NF-κB* and especially *IRF3* and subsequent production of Type-I-IFNs. Interestingly, this induction of IFNs does not necessarily require viral transcription. Instead, in human respiratory epithelial cell models such as A549 cells, the mere interaction of the viral fiber protein with the host cell receptor CAR is sufficient to trigger early inflammatory responses within the first 10 min after receptor binding, including induction of the transcription and regulatory factors *ERK1/2*, *JNK*, *MAPK,* and *NF-κB* (7, 51–54). By contrast, no comparable innate immune activation was detected when A549 respiratory epithelial cells were exposed to purified adenoviral penton or hexon proteins instead of the fiber protein, suggesting that, at least in these transformed airway epithelial cell models, CAR-fiber engagement rather than other capsid components is the dominant trigger of this very early inflammatory response (51, 52).

Furthermore, only the interaction between the adenoviral fiber and the CAR receptor, but not between the fiber and heparan sulfate proteoglycans, was found to promote the transcription of chemokines such as interleukin-8, GRO-α, GRO-γ, RANTES, and interferon-inducible protein 10 (51). Consequently, in A549 and related epithelial cell models, the studied HAdV-B types that predominantly use CD46 or DSG-2 rather than CAR differ from CAR-binding adenovirus types in their early receptor-dependent signaling events and do not induce detectable IFN expression solely via receptor-fiber interaction (43, 55). In these systems, viral DNA appears to be required for the first inflammatory response (55). However, whether similar differences apply to primary respiratory epithelia or immune cells has not yet been systematically addressed.

Upon entry via the CAR receptor, representative species C viruses such as HAdV-C2 and HAdV-C5 are swiftly released into the cytoplasm through early endosomes in human epithelial cell models. Conversely, after entry via CD46, representative species B viruses (HAdV-B3 and HAdV-B35) tend to associate with late endosomes and lysosomes. In this context, TLR9, located on the endosomal membranes, and MyD88-dependent mechanisms seem to play a crucial role in the first immune response induction in these model systems (56–58).

## IMMUNE RESPONSES TRIGGERED BY ADENOVIRUSES ON THEIR WAY THROUGH THE CYTOPLASM

**Fig 2:**
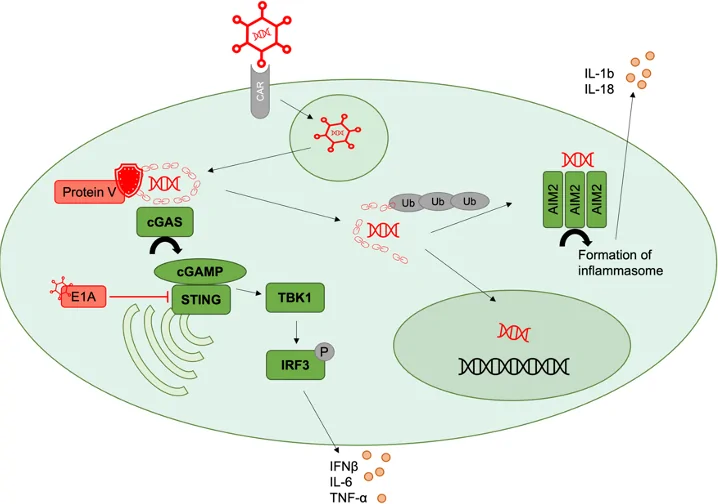
Schematic illustration of immune responses triggered by adenoviruses on their way through the cytoplasm. Upon detection of cytosolic viral DNA, cGAS undergoes conformational changes and synthesizes the second messenger cGAMP. cGAMP binds to the adapter protein STING, leading to activation of TBK1 and phosphorylation of IRF3. This signaling cascade induces the production of Type-I-IFNs and proinflammatory cytokines (60–67). Adenoviral genomes can also be recognized by the cytosolic DNA sensor AIM2, triggering AIM2-dependent inflammasome formation, activation of caspase-1, and secretion of interleukins in professional antigen-presenting cells (68, 69). Adenoviruses counteract DNA sensing through several mechanisms: the viral protein V shields the viral genome from cGAS recognition in the cytoplasm, and the viral E1A protein interacts with STING to inhibit host DNA sensing. For nuclear import of the adenoviral dsDNA, protein V must be removed from the genome via ubiquitination (70, 71). CAR = coxsackie and adenovirus receptor, cGAS = cyclic GMP-AMP synthase, cGAMP = cyclic guanine adenine monophosphate, STING = stimulator of interferon genes, TBK1 = tank-binding kinase 1, IRF3 = interferon regulatory factor 3, IFN = interferon, IL = interleukin, TNF = tumor necrosis factor, Ub = ubiquitination, and AIM2 = absent in melanoma 2.

Whenever HAdVs manage to escape the endosomes, the DNA sensor cGAS plays a crucial role in recognizing adenoviral dsDNA in the cytosol (59). Upon sensing foreign DNA, cGAS undergoes conformational changes, homodimerizes, and synthesizes the messenger molecule 2′−3′ cyclic guanine adenine monophosphate (cGAMP). CGAMP then binds to its adapter protein STING, subsequently activating tank-binding kinase 1 (TBK1) and leading to phosphorylation of IRF3. This cascade results in the secretion of large amounts of Type-I-IFNs, particularly IFNβ, as well as secretion of modest levels of IL-6 and TNF-α (Fig. 2). Notably, cGAS and STING are indispensable for initiating the anti-adenoviral response in antigen-presenting cells (APCs) (60–67).

Since cGAS functions as a cytosolic sensor of nucleic acids, it requires free dsDNA within the cytosol to be triggered. Previous research in human cell models has demonstrated that adenoviruses such as HAdV-C5 protect their genome from cGAS detection while crossing the cytoplasm within the protein capsid, particularly with the aid of the core protein V (70). For nuclear import of the adenoviral dsDNA, detachment of protein V from the genome occurs via ubiquitination, which relies on the E3 ubiquitin ligase Mib1 and the proteasome. Thus, in these systems, intact protein V prevents exposure of free adenoviral dsDNA in the cytoplasm and thereby limits activation of the cGAS-STING pathway. Given that protein V is highly conserved across more than 100 human HAdV types, this shielding mechanism is likely broadly conserved, although it has so far been functionally dissected in detail mainly for HAdV-C5 (70), (Fig. 2). Specifically, it was observed that during infection of macrophage-like MPI-2 cells with an HAdV-C5 variant lacking protein V, the infected cells secreted significantly higher levels of cytokines such as IFNβ than during wild-type HAdV-C5 infection, which highlights the protective function of the viral protein V (70).

Adenoviral types entering host cells via CAR (HAdV-C2 and -C5) as well as types mainly entering via DSG-2 (HAdV-B7) or CD46 (HAdV-B35) both activated the cGAS-STING pathway in immortalized human cell lines (72). However, the type-specific entry, endosomal escape, and viral DNA protection mechanisms seemed to influence the extent of cGAS activation, with HAdV-B7 and HAdV-B35 inducing stronger cGAS activation than HAdV-C2 and HAdV-C5 in these cell models (72). Furthermore, it was shown that the adenoviral E1A protein interacts with STING, leading to inhibition of host DNA sensing by the virus (71). These mechanistic insights predominantly originate from studies in immortalized human cell lines and professional APCs, most often using HAdV-C5, HAdV-C2, HAdV-B7, or HAdV-B35 types. It remains unclear to what extent cGAS-STING activation and its antagonism are conserved across other HAdV species in primary epithelial cells or tissue-relevant organoids.

In addition, HAdV genomes were shown to directly interact with the cytosolic DNA sensor AIM2, which triggers AIM2-dependent inflammasome formation, activation of caspase-1, and secretion of IL-1β in human professional antigen-presenting cells such as monocyte-derived dendritic cells and macrophages *in vitro* (68, 69) (Fig. 2).

Furthermore, antimicrobial peptides such as defensins, produced by epithelial and immune cells, have been shown to inhibit viral infections by interfering with viral entry or replication and modulating host cell signaling pathways (73). Specifically, α-defensins inhibit adenovirus infection in cell culture through direct binding to the virion. By thereby stabilizing the capsid, they prevent disassembly at the vertex region and the release of the internal capsid protein pVI, which is required for endosomal escape. Consequently, virions remain trapped in endosomal compartments (74). Interestingly, the sensitivity of adenoviruses to α-defensins was found to be species-dependent in such neutralization assays. While representatives of species A, B, C, and E are generally susceptible to defensin-mediated neutralization, adenoviruses from species D and F are largely resistant. These differences are thought to be determined by structural variations in capsid proteins such as the fiber and penton base, which influence defensin binding and, therefore, capsid stability (75, 76).

## NUCLEAR IMMUNE DEFENSE MECHANISMS AGAINST ADENOVIRAL INFECTIONS

Once the viral genome has entered the nucleus, host cells deploy multiple intrinsic antiviral defense mechanisms that restrict viral gene expression and replication. These nuclear antiviral responses include chromatin-based restriction, DNA damage response (DDR) signaling, and PML nuclear body-mediated antiviral activity, which are targeted by numerous DNA viruses to promote productive infection (77, 78).

**Fig 3:**
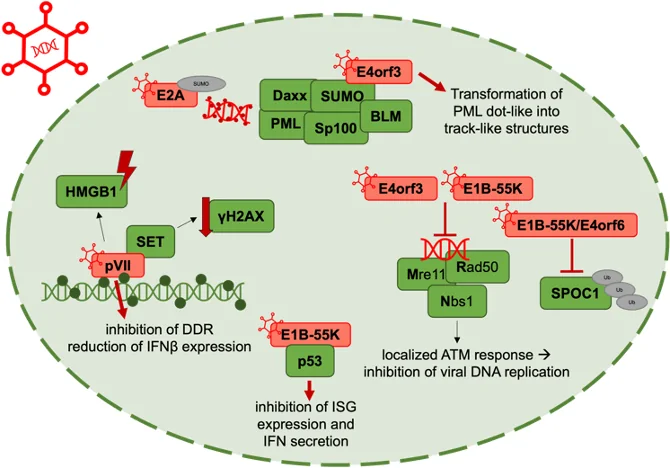
Schematic illustration of nuclear immune defense mechanisms against adenoviral infections. After entry of the adenoviral genome into the nucleus, several host defense pathways are activated that restrict viral replication. The viral protein VII associates with host nucleosomes and sequesters HMGB1, thereby limiting danger signaling and reducing γH2AX accumulation. Through interaction with the host factor SET, this leads to suppression of DDR and reduced IFNβ expression (79–81). The host MRN complex can recognize viral DNA and initiate a localized ATM-dependent DDR that inhibits viral DNA replication. HAdVs counteract this response through the viral proteins E4orf3 and E1B-55K. E4orf3 induces relocalization of MRN components, while the E1B-55K/E4orf6 E3 ubiquitin ligase promotes degradation of antiviral factors such as SPOC1. In addition, E1B-55K interferes with p53 signaling, thereby reducing ISG expression and IFN secretion (84–90). PML-NBs contain proteins such as PML, Daxx, Sp100, BLM, and SUMO. During early infection, the HAdV-C5 protein E4orf3 reorganizes PML-NBs from dot-like structures into elongated track-like structures. This reorganization disrupts PML-NB-mediated antiviral responses and promotes viral replication (91–95). HMGB1 = high-mobility-group-protein 1, SET = SET nuclear proto-oncogene protein, DDR = DNA damage response, IFN = interferon, SUMO = small ubiquitin-related modifier, Daxx = death domain-associated protein, PML = promyelocytic leukemia, BLM = bloom, SPOC1 = survival time-associated PHD (plant homeodomain) finger protein in ovarian cancer 1, Ub = ubiquitination, ATM = ataxia telangiectasia mutated, ISG = interferon-stimulated genes, Sp100 = speckled protein of 100 kDa, and γH2AX = phosphorylated histone H2A.X.

Within this context, studies using HAdV-C5 infection of immortalized human cell lines and *in vivo* models demonstrated that the core protein VII sequesters HMGB1, a host protein released in response to inflammation, serving as a danger signal to activate immune response (79) (Fig. 3). Consequently, studies showed that the presence of protein VII leads to a reduction in IFNβ expression (80). This effect appears to be specific to IFNβ as the transcription of downstream ISGs remained unaffected (80). Additionally, protein VII interacts with the host oncoprotein SET, resulting in a significant reduction in γH2AX accumulation and ultimately leading to the inhibition of DDR signaling (81) (Fig. 3). Again, most of these data were acquired through studies on HAdV-C5. Although protein VII is highly conserved and similar effects have been reported for selected types such as HAdV-C5, HAdV-D9, and HAdV-A12, comprehensive, side-by-side comparisons across species and in primary human cell models are still lacking. Thus, functions inferred as “conserved” should be regarded as working hypotheses rather than fully validated pan-adenoviral mechanisms (79, 80, 82, 83).

The host DNA repair machinery within the nucleus has developed mechanisms to neutralize viral genomes without jeopardizing cellular viability and growth. The cellular DDR constitutes an intricate network of signaling pathways, most prominently ATR and ATM signaling, to protect cellular DNA, ensuring genomic integrity during replication (96, 97). In response to damaged DNA lesions such as DNA double-strand breaks (DSBs) or accumulation of single-stranded DNA (ssDNA), DDR is activated. This activation leads to the recruitment of cellular factors for lesion repair or the induction of cell cycle arrest (97). This process involves recruitment of cellular factors such as the MRN complex (consisting of Mre11, Rad50, and Nbs1) to repair the DNA lesions and halt cellular replication (97).

For antiviral defense, the MRN complex can also bind to the adenoviral genome and initiate a localized ATM response that specifically inhibits viral DNA replication while leaving cellular replication unaffected (67, 84). Notably, ATM activation triggered by viral genomes differs from that induced by chromosomal DSBs as it does not involve the chromatin modifications mediated by phosphorylated H2AX (γH2AX), which are typically required for full DDR activation (84). This distinction was, however, primarily characterized in HAdV-C5-based systems and awaits confirmation across other types and in primary cell contexts.

Manipulation of the DDR is a common strategy used by many DNA viruses, including adenoviruses and herpesviruses, to facilitate viral replication while avoiding antiviral restriction mechanisms (98). This host antiviral defense mechanism was shown to be counteracted by the adenoviral proteins E1B-55K and E4orf3, which inhibit the MRN-ATM DDR complex (Fig. 3). Additionally, the adenoviral E1B-55K/E4orf6 ubiquitin ligase complex facilitates proteasomal degradation of the antiviral DDR factor SPOC1 (84–86). The E1B-55K protein also inhibits the induction of ISGs by interacting with p53 and thereby their positive feedback on IFN secretion (87–90) (Fig. 3).

These findings were primarily derived from detailed studies on HAdV species C and were largely obtained in transformed cell lines such as HeLa, A549, and H1299. A notable exception is the E1B-55K-dependent repression of ISGs, which was also demonstrated in normal human fibroblasts and respiratory epithelial cells (89). However, a comprehensive review examining DDR proteins targeted by various HAdV types highlighted significant differences between DDR targeting strategies and the effectiveness of different types (99). For example, E1B-55K from types A12, C5, E4, and F40, in combination with E4orf6, facilitates the degradation of the DDR component Mre11. In contrast, types B3, B7, B11, B34, and D9 do not exhibit this activity (85, 99–102). Therefore, adenovirus types show varying degrees of susceptibility to inhibition by the MRN complex. HAdV-C2, -C5, and -E4 were able to inactivate the MRN complex and, consequently, their replication remained unaffected by DDR, whereas the replication of HAdV-A12 and -D9 was hindered, although they employed strategies such as mislocalization or degradation to target MRN. It should be noted that these comparative analyses were likewise conducted predominantly in transformed human cell lines (HeLa and A549), so the extent to which these type-specific hierarchies hold in primary epithelial cells or organoid models remains to be established. This indicates that these mechanisms alone were insufficient to fully counteract the inhibitory effects of the MRN complex on viral DNA replication (99, 103).

Furthermore, PML-NBs, dynamic multiprotein dot-like structures, play a crucial role as nucleus host antiviral mechanisms (104, 105). The primary structural elements within PML-NBs encompass the tumor suppressor PML, the chromatin remodeler Daxx, the transcriptional regulator Sp100, the Bloom helicase (BLM), and the small ubiquitin-like modifier (SUMO) (106, 107). Promoted by SUMOylation of the adenoviral DBP E2A, HAdV replication centers are formed juxtaposed to those PML-NBs (108) (Fig. 3).

Up to this point, seven PML isoforms are known. Their transcription is stimulated by IFNα, β, and γ via IFNα-/β-stimulated responsive elements (ISRE), IFNγ-stimulated activation sites (GAS), and by p53 (109–111). Driven through this IFN stimulation, PML-NBs are strongly involved in antiviral defense mechanisms, whereby many DNA viruses such as herpesviruses and adenoviruses have evolved mechanisms to avoid this cellular defense possibility and exploit it for enhancement of their own viral replication and progeny production (112–115). Studies on herpesviruses have provided important insights into the molecular mechanisms by which nuclear antiviral structures and interferon-mediated responses are targeted during infection, highlighting the broader relevance of these pathways for host-virus interactions (116, 117).

During initiation of viral replication, E4orf3 of HAdV-C5 interacts with and reorganizes PML-NBs, specifically the PML-II isoform, by inducing a transformation from their characteristic dot-like structures into elongated, track-like structures (91–94). This E4orf3-driven reorganization antagonizes the PML-NB-induced, especially PML- and Daxx-induced, innate antiviral response (95). Host factors such as Sp100A and PML-II, in combination with viral E1A, promote viral replication and colocalize with those track-like structures. In contrast, inhibitory factors such as Daxx, ATRX, and p53 are either proteasomally degraded via ubiquitin ligation mediated by E1B-55K- or E1B-55K-/E4orf6-induced E3 ubiquitin ligase or via proteolysis through the SUMO-dependent ubiquitin ligase RNF4 (118–123) (Fig. 3).

Additionally, the antiviral PML-NB components Sp100 B, Sp100 C, and HMG are actively relocalized from PML-NBs and sequestered to viral replication centers, thereby neutralizing their host restriction functions (119). Research also demonstrated that various splice variants of PML can either facilitate or hinder the adenoviral infection cycle. PML-I and PML-II contribute to the formation of HAdV-induced track-like structures and the establishment of viral replication centers. Conversely, PML-III, -IV, -V, and -VI inhibit viral gene expression and progeny production (91, 123). It should be emphasized that these mechanistic data on PML-NB reorganization and the targeting of Daxx, Sp100, and PML isoforms were obtained almost exclusively with HAdV-C5 in transformed or immortalized human cell lines (H1299, HeLa, U2OS, and HepaRG), while the contribution of E1B-55K to oncogenic transformation was assessed in primary rodent cells. Corresponding data in primary human epithelial cells or organoids are currently lacking. Based on sequence comparisons and molecular phylogenetic analyses of the adenoviral species in prior literature, it was hypothesized that the E4orf3 of species C exhibits significant differences from those of other HAdV species (124). However, it was demonstrated that, in addition to HAdV-C5, infection with types HAdV-E4 and HAdV-A12 also leads to E4orf3-dependent PML relocalization into track-like structures in HeLa and A549 cells (124). As this cross-type evidence is restricted to the E4orf3-mediated relocalization of PML and was again established in transformed cell lines, further studies are needed to elucidate if this is a conserved function across all HAdV species and whether it operates comparably in physiologically relevant primary cell contexts (124).

## IFN SIGNALING IN HUMAN INNATE IMMUNE RESPONSES TO ADENOVIRAL INFECTION

If adenoviruses fail to counteract host immune responses, innate immune responses lead to the upregulation of IFN production and secretion, ultimately suppressing adenoviral infection. This suppressive effect of host IFN signaling on adenoviral infection is partially attributed to an IFN-responsive inhibitory element located within the enhancer region of the adenoviral E1A gene (125). Both IFNα and IFNγ inhibit the interaction between the transactivator of viral gene expression GABP and the E1A enhancer region early in infection. At the same time, IFNs also enhance the binding of the E2F-associated inhibitory pocket proteins, Rb and p107, to the E2F binding site within the E1A enhancer (125).

**Fig 4:**
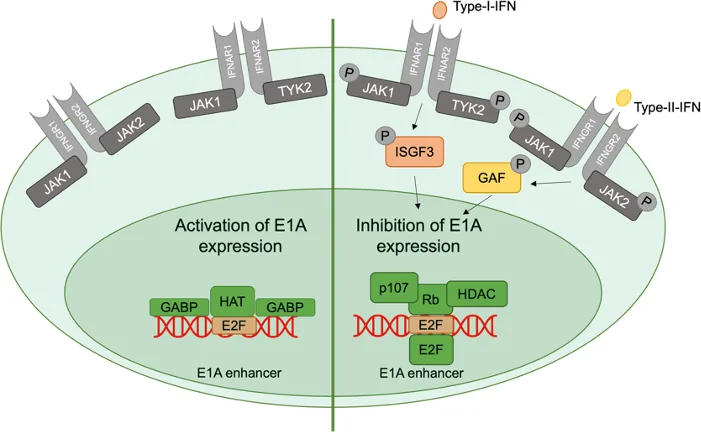
IFN-mediated suppression of adenoviral infection. In the absence of IFN signaling, the viral E1A enhancer is transcriptionally active. Transcription factors such as GABP and HAT bind to the E1A enhancer region together with E2F, promoting chromatin accessibility and activation of E1A transcription. Following Type-I/II-IFN signaling, activation of the JAK-STAT pathway results in phosphorylation of STAT proteins and formation of the transcription factor complexes ISGF3 or GAF. These complexes trigger antiviral gene expression and lead to repression of the E1A enhancer. Mechanistically, IFN signaling promotes displacement of activating factors such as GABP and HAT and recruitment of pocket proteins including Rb and p107, together with HDACs, resulting in inhibition of E1A transcription (125). IFN = interferon, ISGF3 = interferon-stimulated gene factor 3, GAS = gamma interferon-activated site, JAK = janus-activated kinase, TYK = tyrosine kinase, IFNAR1/2 = IFNα receptor 1/2, IFNGR1/2 = IFNγ receptor 1/2, GABP = GA-binding protein, HAT = histone acetyltransferase, Rb = retinoblastoma tumor suppressor, HDAC = histone deacetylase.

Together, these mechanisms suppress E1A gene expression and subsequently activation of viral DNA replication and efficient adenoviral infection. However, if the virus manages to express the viral E1A protein by counteracting the host immune response, this leads to disrupted interactions between E2F and pocket proteins, restoring HAdV DNA replication in the presence of IFNα or IFNγ (125) (Fig. 4).

Interestingly, research investigating the mechanisms of IFN-induced establishment of persistent adenoviral infections, including inhibition of GABP binding to the E1A enhancer, association of Rb family proteins and HDAC within the E1A enhancer region, and downregulation of adenoviral transcription by IRE1α nuclease, has also been carried out in the context of HAdV-C5 infection (125–127).

However, it has been demonstrated that the involved GABP- and E2F-binding sites in the E1A enhancer regions of the viral genomes are conserved across adenoviral species A, B, D, and E. Sequence comparisons between the E1A genes of species F revealed a high degree of homology with the other species in GABP- and E2F-binding sites despite their 3′ binding site for E2F (128). Nevertheless, enteric adenoviruses might also be susceptible to IFN through binding to upstream sites in the E1A enhancer, but whether a similar mechanism is active during species F virus infection remains unknown (128).

HAdV types vary in their ability to counteract host immunity and their potential to prevent host IFN signaling. The most detailed studies were conducted based on HAdV-C5. For HAdV-C5, E1A has been shown to inhibit IFN signaling in cell line models by directly binding essential STAT proteins, thereby preventing the formation of ISGF3 and GAF complexes and the activation of nucleic acid sensing pathways possibly detecting adenoviral DNA in the cytosol (129–131). HAdV-C5 sequesters also phosphorylated STAT1 proteins at viral replication centers and inhibits STAT dephosphorylation, thereby counteracting the active form of STAT1 (132). Comparable mechanistic data for other HAdV species or for primary epithelial and immune cells are largely missing, and it is conceivable that E1A-STAT interactions and their impact on IFN signaling may differ between types.

In addition to its interaction with STAT, E1A also binds to other host regulatory proteins such as FoxK, DCAF7, and RuvBL1, leading to the suppression of Type-I-IFN signaling (133, 134).

Another E1A-mediated strategy in this context involves altering posttranslational modifications of cellular histone proteins, catalyzed by the hBre1/RNF20 complex and necessary for the activation of ISGs in response to viruses. The HAdV-C5 E1A protein binds to and disassociates the hBre1 complex, a ubiquitin ligase, thereby blocking IFN-induced H2B monoubiquitylation and the dependent ISG expression (135).

In addition, E1A utilizes hBre1 as a scaffold protein to recruit hPaf1. Consequently, E1A activates viral early transcription, initiating viral replication (136). E1A further impedes the induction of HLA class II genes activated by IFNβ and IFNγ (130, 137).

The E1A gene is exclusive to the genus *Mastadenovirus* and is largely conserved across different mastadenoviral types (1). However, certain regions exhibit variability, such as IDR23, which contributes to E1A’s transforming activity, IDR34, which plays a role in transcriptional regulation, and IDR12, which is involved in host protein interactions (138). This variability suggests that some molecular functions of E1A may have evolved, reflecting adaptations over time and resulting in different levels of oncogenicity and resilience to the host immune system of different types (138). For HAdV-F41, it has been shown that its E1A products exhibit a reduced ability to transactivate other viral early genes and consequently promote efficient viral DNA replication (128, 139).

Among other things, host *IFNβ* expression is triggered by the presence of adenoviral dsRNA sequences, which arise from symmetrical transcription processes occurring in the late phase of infection. Upon detection, those dsRNA sequences initiate a signaling cascade that culminates in the translocation of IRF3 and NFκB into the nucleus, where they act as transcription factors to promote the expression of *IFNβ*. The E1A protein of HAdV-C2 and HAdV-C5 is known to interfere with the IRF3 activity by binding to p300/CBP, thereby inhibiting *IFNβ* expression (140–142). However, the E1A genes of HAdV-C2 and HAdV-C5 compared to HAdV-A12 exhibit differences in their p300 binding capacity, contributing to their different IFN expression patterns (140).

Adenoviral dsRNA sequences can also be detected by the host protein kinase R (PKR), resulting in the phosphorylation of the eIF2α translation factor and ultimately in the inhibition of late viral protein translation. However, some HAdV species are able to inhibit the host PKR through their virus-encoded RNA I (VA RNA I) (143, 144). For example, HAdV-C types such as HAdV-C2 efficiently suppress PKR activity through VA RNA I expression, thereby allowing continued viral protein translation. In contrast, HAdV-A12 produces substantially lower levels of VA RNA I, resulting in incomplete inhibition of PKR and reduced late protein synthesis. Consistent with this, introducing HAdV-C2 VA RNA I into cells enhanced HAdV-A12 hexon protein expression, indicating that limited VA RNA I production of HAdV-A12 may play a role in its restricted replication (143). These conclusions are primarily based on comparative studies of HAdV-C2 versus HAdV-A12 in transformed human cells, and their generalizability to other species, types, or primary cells remains to be determined.

**Fig 5:**
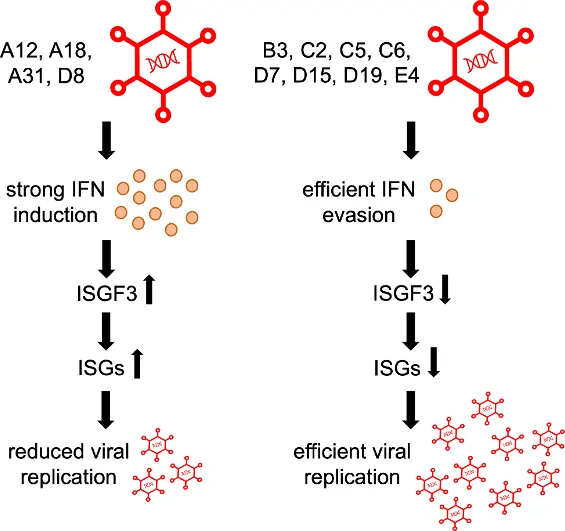
Type-specific differences in IFN responses during HAdV infection. HAdV types differ in their ability to induce or evade host IFN responses. Certain types, such as HAdV-A12, -A18, -A31, and -D8, strongly activate IFN signaling, resulting in increased IFNβ expression and activation of the ISGF3 transcription factor complex, which induces expression of ISGs and establishes an antiviral state that restricts viral replication. In contrast, other types such as HAdV-C2, -C5, -C6, -B3, -E4, -D7, -D15, and -D19 efficiently suppress IFN signaling, resulting in only transient IFNβ induction and minimal ISG expression, thereby enabling efficient viral replication (140, 143, 145–148). IFN = interferon, ISGF3 = interferon-stimulated gene factor 3, and ISG = IFN-stimulated gene.

Some studies focused on the type-specific potential to induce or evade host IFN signaling. HAdV types D8, A12, A18, and A31 were more potent inducers of IFN responses compared to C2, B3, E4, C5, C6, D7, D15, and D19 in chick embryo cells, which may not fully reflect human primary target cells (145), (Fig. 5). In more detail, one study showed that HAdV-A12 is unable to evade host IFN-mediated immunity to the same extent as HAdV-C2, which might explain their different pathogenicity and course of infection. HAdV-A12 is characterized by a slow course of infection with relatively mild cytopathic effects on host cells. However, it exhibits pronounced oncogenic potential (140, 146, 147). During A12 infection, mRNA levels of *IFNB*, several ISGs, and all components of the ISGF3 transcription factor *STAT1*, *STAT2,* and *IRF9*, crucial for the IFN signaling pathway, were upregulated after infection, indicating an activated host immune response to the infection (140, 146, 147) (Fig. 5). In contrast, none of these ISGs were induced during infection with HAdV-C2, and *IFNβ* expression was only transiently elevated (140, 143). Systematic comparative studies in human primary epithelial cells, organoids, and APCs are needed to validate whether these type-specific IFN induction patterns hold in more physiological settings.

Experimental studies focusing on selected species B types suggest that some HAdV-B viruses (HAdV-B7 and -B14) can exhibit a pronounced resistance to Type-I-IFNs in transformed epithelial cell models, likely due to efficient suppression of ISG expression by blocking nuclear translocation of host regulators such as RuvBL1/2 (149). However, current data are limited to specific B-types and cell lines, so these findings should be interpreted as type- and context-dependent rather than as a universal property of all HAdV-B viruses.

HAdV-F40 and -F41 infection efficiencies compared to HAdV-C2 were reduced by IFN treatment in cancerous Chang conjunctival cells. However, when cells were infected with HAdV-C2 prior to F41 infection, it rescued HAdV-F41 from the effects of IFN. This suggests that HAdV-C2 effectively suppressed the antiviral state induced by IFNs, while F41 alone did not have the same capability (150). Experimental data confirmed that during HAdV-C5 and -C6 infection, secretion of IFNα, β, and λ was not significantly increased in comparison to the uninfected control in A549 cells, while *IFNB* and *IFNL* mRNA levels slightly increased (148).

Most mechanistic insights into adenovirus-induced innate immune responses have been obtained from *in vitro* studies. Studying HAdV infections *in vivo* remains challenging because most laboratory animals are not natural HAdV hosts and, therefore, do not fully reproduce human infection. Classical animal models often show limited viral replication or fail to develop characteristic disease symptoms (151). To overcome these limitations, transgenic mouse models expressing HAdV receptors such as CAR, CD46, or DSG-2 have been developed and allow improved investigation of viral replication and host immune responses (152–154). In addition, human primary cell cultures and organoid systems have emerged as promising models to study adenovirus-host interactions under more physiologically relevant conditions (155, 156). However, these systems still require further validation and standardization.

Collectively, many of the mechanistic insights into HAdV IFN evasion and modulation have been gained in a relatively narrow range of transformed human cell lines, such as A549 or HeLa-derived cells, and in specialized immune cells. These models have been invaluable for dissecting pathways such as cGAS-STING activation, E1A-STAT interactions, or PKR inhibition, but they only partially recapitulate the biology of primary respiratory or intestinal epithelia, tissue-relevant organoids, and *in vivo* immune environments. Future studies that systematically compare multiple HAdV species and types across primary epithelial cells, organoid systems, and professional immune cells will be essential to distinguish truly generalizable adenovirus biology from cell line- or cell type-specific phenomena.

## IMPLICATIONS OF ADENOVIRUS-HOST IMMUNE INTERACTIONS FOR ADENOVIRUS-BASED VECTOR DESIGN

In addition to their role in viral pathogenesis, adenovirus-host immune interactions have important implications for the development and application of adenovirus-based vectors. Several adenoviral mechanisms that modulate host immune responses are encoded within the E3 region of the viral genome (130, 157, 158). Notably, the E3 region exhibits substantial genetic variability among different HAdV species. For example, it spans approximately 5.2 kb in species D but only about 3 kb in species F (159). In this context, a species D-specific E3 protein, E3/49K, has been identified. In contrast to most other E3 proteins, which primarily act on infected cells, E3/49K appears to target uninfected cells. After secretion, E3/49K can inhibit the activation and cytotoxic function of natural killer cells, as well as the activation, signaling, and cytokine production of T cells (160). To date, the specific function of the HAdV-F E3 genes remains unknown. However, it is hypothesized that these unique E3 proteins may also aid the virus in evading immune surveillance by natural killer cells within the infected gut (157).

These variations in virus-host interactions may also influence the behavior of adenovirus-based vectors *in vivo*. Structural differences between adenovirus types can affect vector biodistribution and immune recognition. For example, HAdV-C6 differs from the commonly used type C5 in its hexon capsid protein. These differences reduce uptake by liver macrophages and alter opsonization by immunoglobulins and other blood proteins. Thereby, they significantly influence the systemic biodistribution of HAdV-C6 following administration (161).

Such properties are particularly relevant for the use of adenoviral vectors in vaccine development. While HAdV-C5 vectors remain the most widely used, their high seroprevalence results in preexisting neutralizing antibodies in many individuals, leading to rapid viral clearance and reduced efficacy during systemic applications. Consequently, alternative adenoviral types with lower seroprevalence are being further explored for vector design (161, 162).

Beyond differences in seroprevalence, adenoviral species- and type-specific interactions with components of the innate and adaptive immune system may further influence the utilization of adenovirus-based vectors and affect their biodistribution and cellular tropism (54, 163, 164). These considerations are illustrated by adenovirus-based vaccine platforms such as the chimpanzee adenovirus-based vaccine vector ChAdOx1 nCoV-19 (AstraZeneca) and the human adenovirus-based vaccine vector Ad26.COV2.S (Johnson & Johnson), which were widely deployed as SARS-CoV-2 vaccines during the COVID-19 pandemic (165). Both vaccines rely on replication-deficient adenoviral vectors that deliver the gene encoding the SARS-CoV-2 spike protein into host cells, where it is expressed and subsequently induces protective immune responses. The vector ChAdOx1 belongs to the Mastadenovirus genus and is closely related to HAdV but benefits from lower levels of preexisting immunity in many human populations (166, 167). A deeper understanding of how different adenovirus genera, species, and types interact with innate immune pathways may, therefore, help guide the selection and design of improved adenovirus-based vectors.

## CONCLUSION AND CLINICAL RELEVANCE

HAdVs typically cause mild and self-limiting infections in immunocompetent healthy individuals (4). However, there are also reports about severe adenoviral infections in immunocompetent patients leading to myocarditis and pneumonia outbreaks in children (168–170). Primarily, HAdV infection manifests as severe and, in some cases, as fatal illness among individuals with compromised immune systems due to congenital, acquired, or mostly drug-induced immunosuppression. Particularly vulnerable groups include recipients of bone marrow and organ transplants, as well as individuals with acquired immunodeficiency syndrome (AIDS). Notably, children after allogenic hematopoietic stem cell transplantation (HSCT) seem to experience HAdV infections more frequently and at an earlier stage in their post-transplant period (4, 171).

Although no specific antiviral therapy has been officially approved for severe adenovirus infections, antiviral agents such as cidofovir are commonly used in immunocompromised patients. In addition, adoptive transfer of virus-specific T cells represents a promising therapeutic strategy for controlling severe adenovirus infections in transplant recipients (172). This emphasizes the need for specific viral treatment options and early diagnostic tools, especially in high-risk patient populations.

As discussed above, HAdVs comprise a diverse range of species and types, exhibiting differences in capsid structure, genome organization, and consequently in their capacity to evade the host immune response. Available data, which are largely derived from *in vitro* studies and limited *in vivo* models, indicate that species such as HAdV-C and certain HAdV-B types can more efficiently counteract host immune responses, whereas enteric types such as HAdV-F40/41 appear to exhibit more limited immune evasion in the studied systems. Whether these differences fully translate to human primary epithelial tissues and immune cells *in vivo* remains to be determined.

In conclusion, a deeper understanding of type-specific immune evasion strategies is critical for understanding the different pathogenicity of adenoviral types, preventing severe adenoviral infections, and also for improving the design and safety of adenovirus-based vectors. In this context, this review underscores the urgent need for broader research beyond HAdV-C5 to unlock the full clinical and biotechnological potential of adenoviruses.
